# Pattern Distribution and Utility of Reporting Anticellular Antibody Over Antinuclear Antibody: An Initial Report From the Immunofluorescence Laboratory of North Bengal Medical College and Hospital, West Bengal

**DOI:** 10.7759/cureus.84262

**Published:** 2025-05-16

**Authors:** Ushasi Banerjee, Arghya Chattopadhyay, Prasenjit Pal, Ivy Banerjee, Avishek Sengupta, Dhiman Sardar, Saheli Mukherjee, Utpal Biswas

**Affiliations:** 1 Department of Biochemistry, North Bengal Medical College and Hospital, Siliguri, IND; 2 Department of Rheumatology, North Bengal Medical College and Hospital, Siliguri, IND; 3 Department of Biochemistry, Sarat Chandra Chattopadhyay Government Medical College, Kolkata, IND

**Keywords:** anticellular antibody (aca), anticytoplasmic antibody, antinuclear antibody (ana), autoantibody, systemic autoimmune rheumatic diseases (sards)

## Abstract

Introduction: Antinuclear antibodies (ANA) are biomarkers used to diagnose, monitor, differentiate, and treat various systemic autoimmune rheumatic diseases (SARDs). The gold standard test for SARD screening involves using human epithelial cells (HEp-2) in an indirect immunofluorescence assay (IIFA) to detect ANA. However, diagnosing autoimmune disorders can be challenging when patients test ANA-negative but exhibit specific autoantibody patterns targeting the cytoplasm or mitotic spindle apparatus in the IIFA. To address this, the International Consensus on Antinuclear Antibody Patterns has proposed the term "anticellular antibodies" (ACA) to encompass various types of autoantibodies, including ANA. This study explores the rationale for shifting from reporting ANA to ACA in diagnosing SARDs.

Materials and methods: An institutional observational study was conducted on 973 suspected and previously diagnosed patients in the Immunofluorescence Laboratory of the Department of Biochemistry of North Bengal Medical College and Hospital, West Bengal. IIFA using HEp-2 and monkey liver tissue (primate) substrates (EUROIMMUN, Lübeck, Germany) was performed after diluting patient serum samples at a ratio of 1:100.

Results: Among the 973 study participants, 585 (60%) tested positive for ACA. Of these ACA-positive cases, 140 (24%) showed anticytoplasmic antibody positivity. This included 38 (6.5%) cases with isolated anticytoplasmic antibody positivity and 102 (17.4%) cases positive for both antinuclear and anticytoplasmic antibody patterns. Isolated anticytoplasmic antibodies were more prevalent among females of reproductive age, accounting for 22 (58%) of such cases.

Conclusions: The IIFA detected various autoantibodies when diagnosing SARDs. The presence of isolated anticytoplasmic antibodies in ANA-negative patients with autoimmune diseases indicates that the term ACA is more appropriate than ANA for diagnosing and reporting SARDs, as it may help reduce false-negative results.

## Introduction

Antinuclear antibodies (ANA) are a specific type of autoantibody produced against various components of the nuclei of the body’s cells, resulting in autoimmune disorders [1]. ANA is an important biomarker for diagnosis, disease monitoring, differential diagnosis, and treatment of various systemic autoimmune rheumatic diseases (SARDs) [2]. A significant increase in the incidence of autoimmune diseases has been observed worldwide over the past decade [3-6].

In the early stages of research, the exact target antigens of these autoantibodies were largely unknown, except for double-stranded DNA and histones. Holman and Robbins coined the term ANA for these antibodies [7,8]. Over the past decade, studies on various SARDs, particularly systemic lupus erythematosus (SLE), have led to the discovery of autoantibodies targeting nuclear components of patients’ cells, establishing the indirect immunofluorescence assay (IIFA) as the standard method for ANA detection [9,10]. Bone marrow examinations of SLE patients revealed the presence of lupus erythematosus (LE) cells containing proteins that exhibit ANA characteristics [11-15]. Various studies have confirmed that the substances found in LE cells of SLE patients represent ANA [16,17].

Over time, multiple methods have been developed for ANA detection in SARD patients. Techniques such as IIFA and solid-phase assay have demonstrated various autoantibody patterns within the cells of SARD patients [18]. The International Consensus on ANA Patterns (ICAP) has taken the initiative to categorize and specify autoantibody patterns beyond ANA in SARD patients. Recently, ICAP highlighted the clinical relevance of 29 distinct HEp-2 IIFA autoantibody patterns [19].

The development of the indirect immunofluorescence method using HEp-2 cells as a standard ANA detection technique has enabled the identification of autoantibodies against additional cellular components, including the cytoplasm, mitotic spindle apparatus, and nuclear membrane [20].

ANA testing using HEp-2 cells in the IIFA and monkey liver primate cells is now considered the gold standard. This dual-substrate approach aids in diagnostic confirmation and measuring titer levels [21]. ICAP has defined specific staining patterns to classify and interpret ANA assays and detailed their clinical relevance [20]. Skilled examiners can precisely identify these staining patterns and titer levels [19]. Because these autoantibodies are detected during ANA diagnostics, ANA has also been applied to them [9].

However, diagnosing autoimmune disorders that are ANA-negative but display specific patterns of autoantibodies against cytoplasmic or mitotic spindle apparatus components in IIFA has led to some confusion. To address this issue, ICAP has proposed the broader term "anticellular antibodies" (ACA), which includes anticytoplasmic antibodies, anti-mitotic spindle apparatus antibodies, and ANA. This terminology aims to expand the diagnostic scope for various SARDs and aid in the early detection of ANA-negative autoimmune disorders [10].

This study aims to support the use of ACA over ANA in the institutional context of North Bengal Medical College and Hospital, West Bengal. The study calculates and analyzes the percentage of patients whose serum samples are negative for ANA but positive for other antibodies, such as those against the cytoplasm and mitotic apparatus.

## Materials and methods

Study population

An institutional observational study was conducted in the Immunofluorescence Laboratory, Department of Biochemistry at North Bengal Medical College and Hospital (NBMC&H), West Bengal, after obtaining approval from the Institutional Ethics Committee of our hospital (approval number: ECR/1701/Inst/WB/2022, approval date: 12th July 2022). The study population included a total of 973 suspected or previously diagnosed subjects with autoimmune disorders from various outpatient and inpatient departments of NBMC&H for two years. The study period encompassed all available records from 2nd May 2022 to 30th April 2024. No sample size calculation was performed since this is an analysis of collected data over a defined time period. The diagnosis was confirmed, and testing was advised by rheumatologists at our hospital. Suspected and previously diagnosed SARD patients of all age groups from various departments who were alive at the time of analysis were included. The exclusion criteria were as follows: patients unwilling to provide blood samples, uncooperative patients, patients with incomplete clinical data, and deceased patients (to prevent survival bias).

ANA and ACA testing

Blood samples from patients with SARDs were collected after completing duly filled checklists. Serum samples were then examined in the immunology laboratory using indirect immunofluorescence (IIF). Signed and completed informed consent forms were obtained from all participants. Data on age, sex, and clinical diagnosis were recorded.

IIFA was performed using HEp-2 cells and monkey liver tissue (primate) substrates (EUROIMMUN, Lübeck, Germany) after diluting the serum samples to a ratio of 1:100. The dilution was carried out using phosphate-buffered saline (PBS). As instructed, the samples were overlaid onto fixed HEp-2 cells and substrates for 30 minutes at room temperature. Slides were washed twice with PBS, followed by applying fluorescence-labeled anti-human globulin and incubation for another 30 minutes. After a second round of PBS washing, the embedding medium was applied and covered with a coverslip.

Two experienced doctors conducted all procedures according to the manufacturer's protocol. Subsequently, the slides were evaluated by four experts using a fluorescence microscope (Eurostar III Plus; EUROIMMUN, Lübeck, Germany), initially at 10× magnification for focusing and then at 40× magnification for observation. ACA was reported as positive if a fluorescent signal was observed at a serum dilution of at least 1:100. Positive antibodies were detected in the nucleus, nucleoli, cytoplasm, spindle apparatus, nuclear membrane, and various other cellular components, with 50% to 70% of the slide area showing fluorescence. The fluorescence patterns were classified according to the ICAP guidelines. Mixed patterns in this study indicated the presence of two or more types of ANA patterns.

Statistical analysis

In this institutional observational study, we have tried to determine the percentage of isolated anticytoplasmic antibody positivity in clinically diagnosed SARD patients who are ANA-negative. We have tried to determine if our findings align with national and international data. Statistical analyses were performed using Microsoft Excel for Windows version 2007 (Microsoft Corp., Redmond, WA, USA) and SPSS Statistics version 22.0 (IBM Corp., Released 2013. IBM SPSS Statistics for Windows, Version 22.0. Armonk, NY: IBM Corp.).

## Results

**Figure 1:**
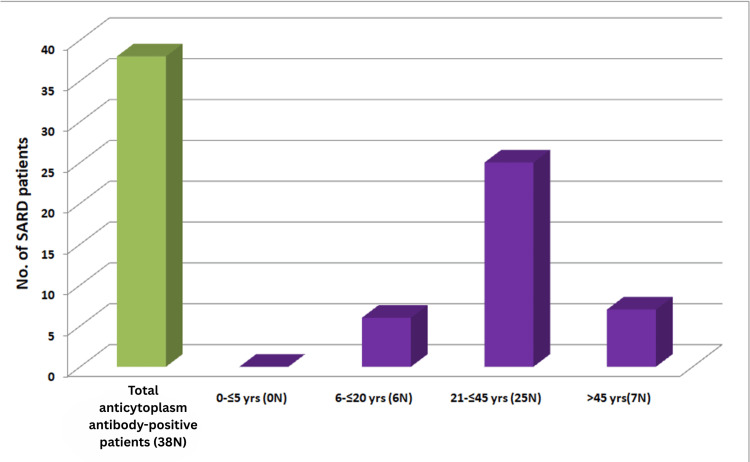
Age distribution of SARD patients positive for isolated anticytoplasmic antibodies (38 (6.5%)) SARD: systemic autoimmune rheumatic disease

**Figure 2:**
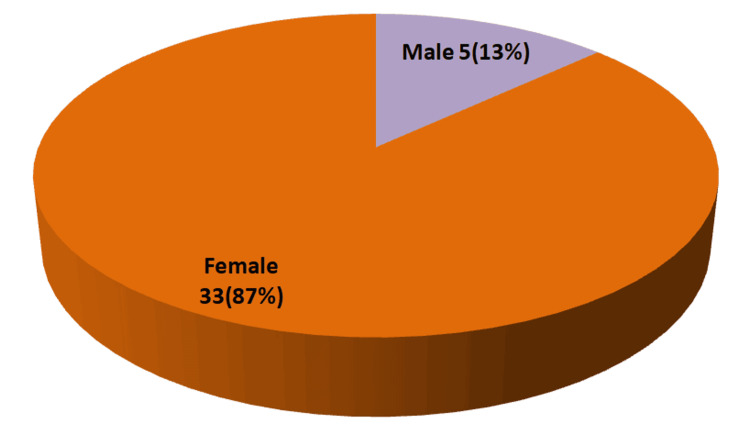
Sex distribution of SARD patients positive for isolated anticytoplasmic antibodies (38 (6.5%)) SARD: systemic autoimmune rheumatic disease

Of 973 suspected or diagnosed autoimmune disease patients, 585 samples tested positive for ACA. This includes cases positive for either ANA or anticytoplasmic antibodies and some cases exhibiting a mixed antibody pattern (citation). The age and sex distribution of isolated anticytoplasmic antibody-positive cases are shown in Figure 1 and Figure 2, respectively.

Figure 1 shows that 65.8% (25 out of 38) of isolated anticytoplasmic antibody-positive cases occur in the reproductive age group (21-45 years). Patients older than 45 years account for 18.4% (7 out of 38), while adolescents represent 16% (6 out of 38) of cases with isolated anticytoplasmic antibody positivity, respectively. Figure 2 demonstrates that isolated anticytoplasmic antibodies are predominantly positive among female SARD patients (33 (87%)).

In Table 1, different patterns of ACA-positive cases are presented. Among the study population, 76% of ACA-positive cases show only ANA positivity, while 6.5% of patients exhibit isolated anticytoplasmic antibodies. Among the ANA-positive SARD patients, the fine speckled pattern is predominant (39.6%). Other ANA-only positive patterns include coarse speckled (19.5%), spindle apparatus (10.1%), homogeneous (6.1%), nucleoli (5.4%), centromere (3.9%), nucleoplasm (2.9%), and nuclear membrane (0.5%), among others. When two or more ANA patterns are observed in the same cell, it is classified as a mixed pattern of ANA (15%).

**Table 1:** Percentage distribution of major ANA and isolated anticytoplasmic antibodies among 585 ACA-positive SARD patients (representing 60% of the total 973 subjects) SARD: systemic autoimmune rheumatic disease, ANA: antinuclear antibodies, ACA: anticellular antibody

Isolated anticytoplasmic antibodies	Total only ANA	Mixed pattern ANA	Homogeneous ANA	Coarse speckled ANA	Fine speckled ANA	Spindle apparatus ANA	Nucleoplasm ANA	Nucleoli ANA	Nuclear membrane ANA	Centromere ANA
38 (6.5%)	445 (76%)	88 (15%)	36 (6.1%)	114 (19.5%)	232 (39.6%)	59 (10.1%)	17 (2.9%)	32 (5.4%)	3 (0.5%)	23 (3.9%)

**Figure 3:**
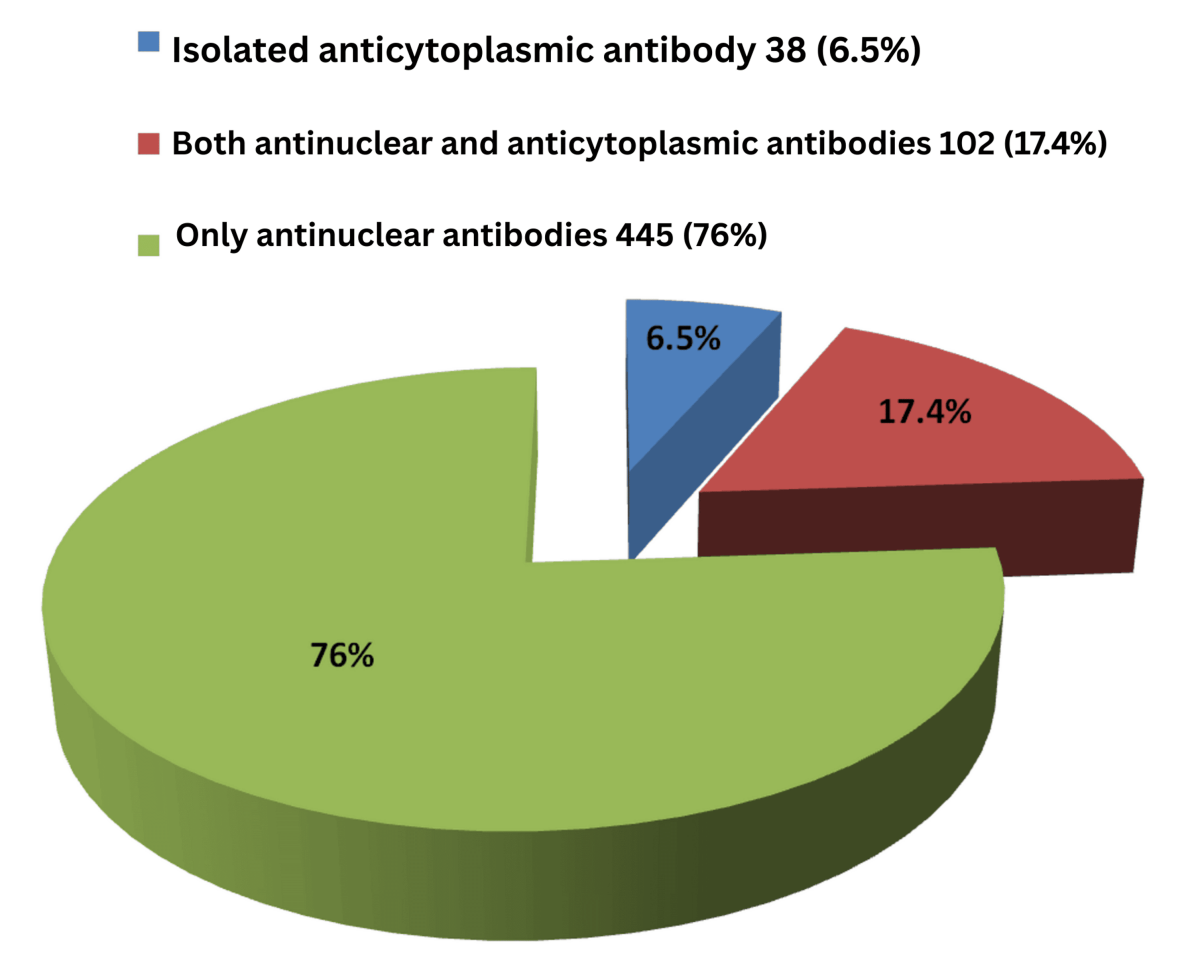
Percentage distribution of ACA positivity among anticytoplasmic antibody-positive SARD cases ACA: anticellular antibody, SARD: systemic autoimmune rheumatic diseases

Figure 3 shows the distribution of total ACA positivity among SARD patients, including 6.5% of cases positive for isolated anticytoplasmic antibodies, 17.4% positive for both ANA and anticytoplasmic antibodies, and the remaining 76% positive only for ANA.

Table 2 depicts the disease associations in isolated anticytoplasmic antibody-positive cases (6.5%). Among these, four (10.5%) show symptoms associated with SLE, five (13%) have vasculitis, three (8%) have Sjögren's syndrome, and another three (8%) have rheumatoid arthritis. Additionally, four (10.5%) are associated with arthritis, five (13%) with connective tissue disorders, and four (10.5%) with liver pathology. Two (5%) exhibit symptoms associated with systemic sclerosis, followed by miscellaneous causes of isolated anticytoplasmic antibody positivity. These miscellaneous causes include chronic diarrhea, polycythemia, interstitial lung diseases, autoimmune hemolytic anemias, etc.

**Table 2:** Disease distribution of isolated anticytoplasmic antibody-positive cases (38 (6.5%)) SLE: systemic lupus erythematosus

SLE	Vasculitis	Sjögren’s syndrome	Rheumatoid arthritis	Arthritis	Connective tissue disorders	Liver pathology	Systemic sclerosis	Miscellaneous
4 (10.5%)	5 (13%)	3 (8%)	3 (8%)	4 (10.5%)	5 (13%)	4 (10.5%)	2 (5%)	8 (21%)

The following figures display distinct antibody staining patterns in primate (monkey) liver cells and the HEp-2 cell line, as observed under an immunofluorescence microscope at 40× magnification.

**Figure 4:**
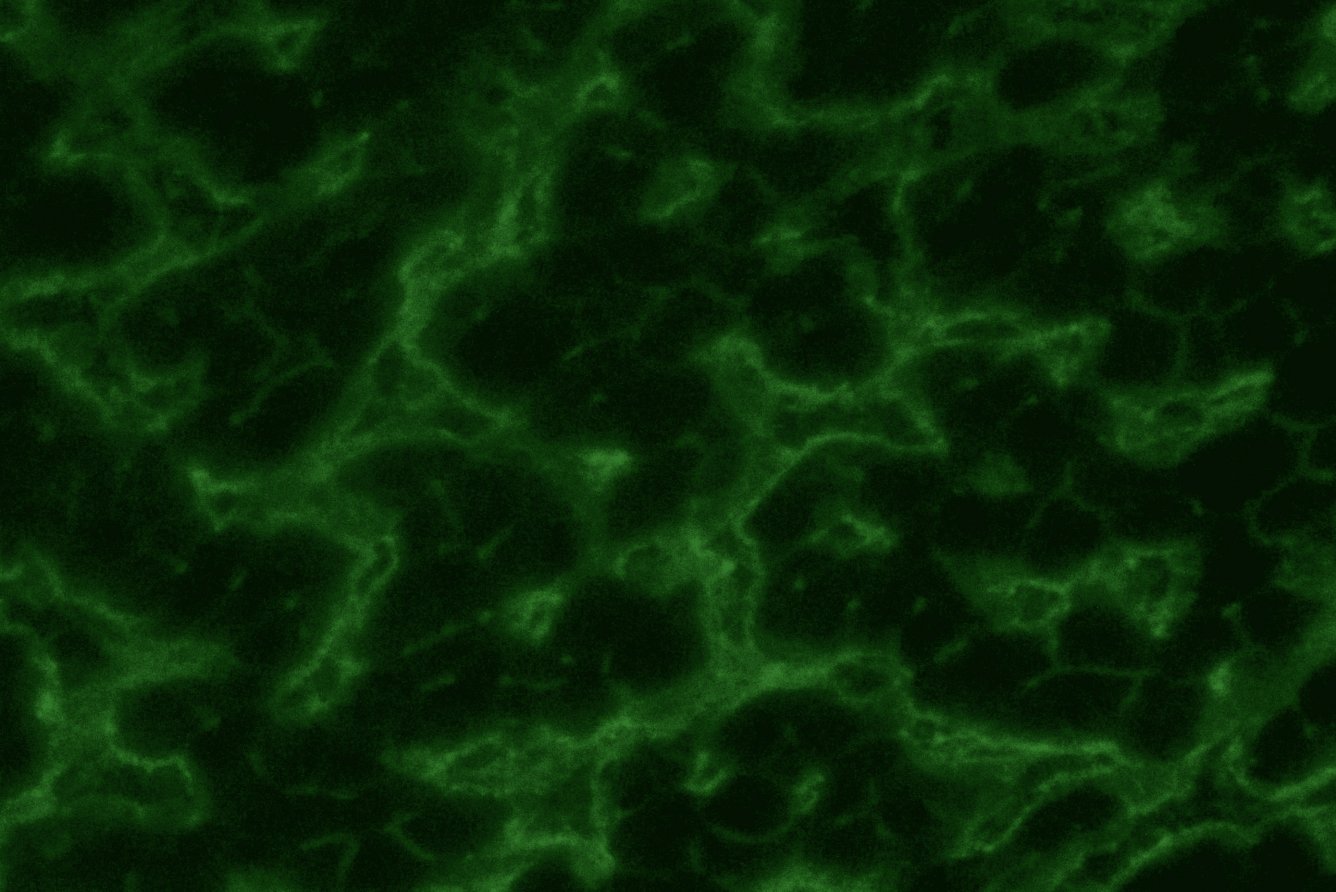
Isolated anticytoplasmic antibodies in primate liver (Monkey) cells under immunofluorescence microscope

Figure 4 shows isolated anticytoplasmic antibodies in primate liver (monkey) cells. No specific staining pattern is observed; a general haziness is present within the cells in the immunofluorescence microscopic field at 40× magnification.

**Figure 5:**
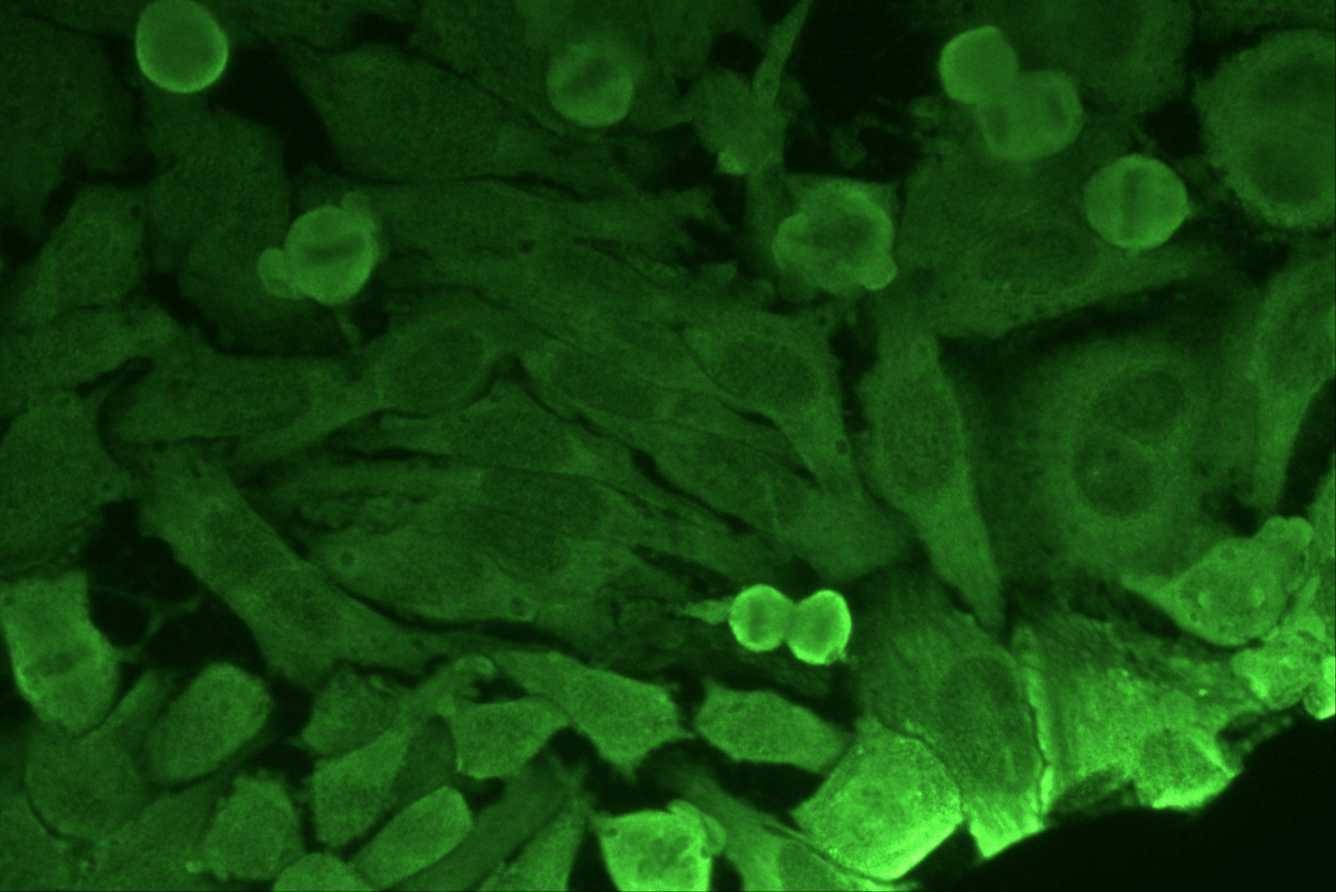
ANA and anticytoplasmic antibody positivity in HEp-2 cell line under immunofluorescence microscope HEp-2: human epithelial cells, ANA: antinuclear antibody

Figure 5 presents isolated anticytoplasmic antibodies and a combination of both ANA and anticytoplasmic antibodies in the cells of the HEp-2 cell line. No fluorescent light comes from the nucleus in the cells with only anticytoplasmic antibodies. Only the cytoplasm of the cells emits fluorescent light. Thus, it appears as if green-colored fluorescence surrounds an oval-shaped dark zone of the cell. There are also some cells in which fluorescent light seems to be emitted from all parts of the cell without distinction between the nucleus and cytoplasm.

**Figure 6:**
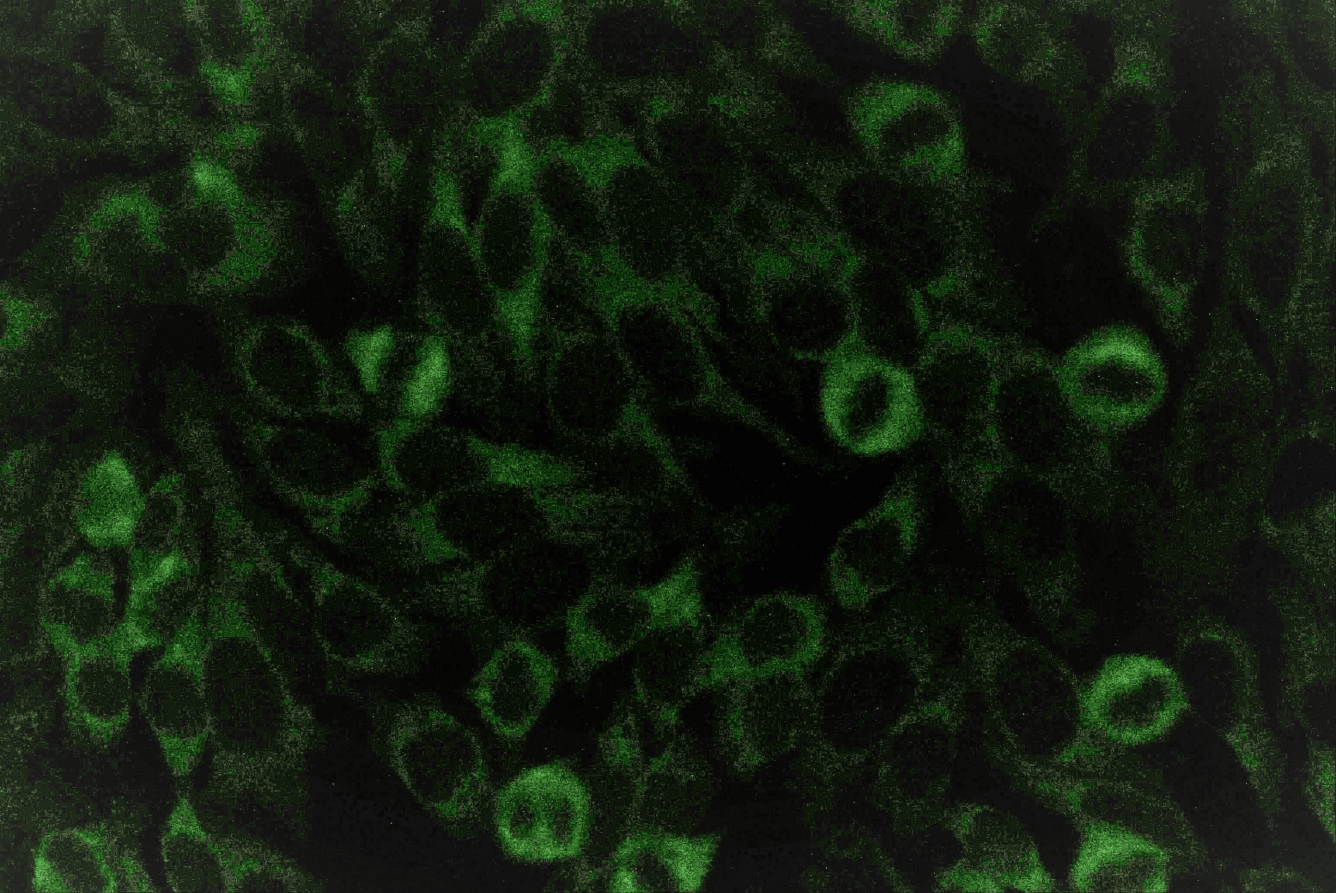
Isolated anticytoplasmic antibody positivity in HEp-2 cell line under immunofluorescence microscope HEp-2: human epithelial cells

Figure 6 shows cells exhibiting only anticytoplasmic antibody positivity. Here, the patient serum contains antibodies directed exclusively against the cytoplasm of the cells identified by the HEp-2 cell line in the immunofluorescence microscopic field. The green fluorescent light emanates solely from the cytoplasm, leaving the cell's nucleus as a dark, black zone.

**Figure 7:**
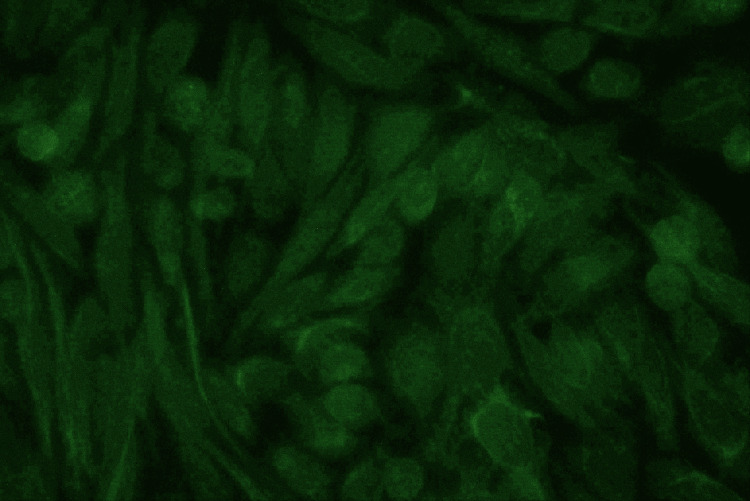
Filamentous pattern of isolated anticytoplasmic antibodies in HEp-2 cell line under immunofluorescence microscope HEp-2: human epithelial cells

Figure 7 shows isolated anticytoplasmic antibodies in the HEp-2 cell line. In some cells, the cytoplasm displays filamentous structures that emit fluorescent light. This is known as the filamentous pattern of anticytoplasmic antibodies.

**Figure 8:**
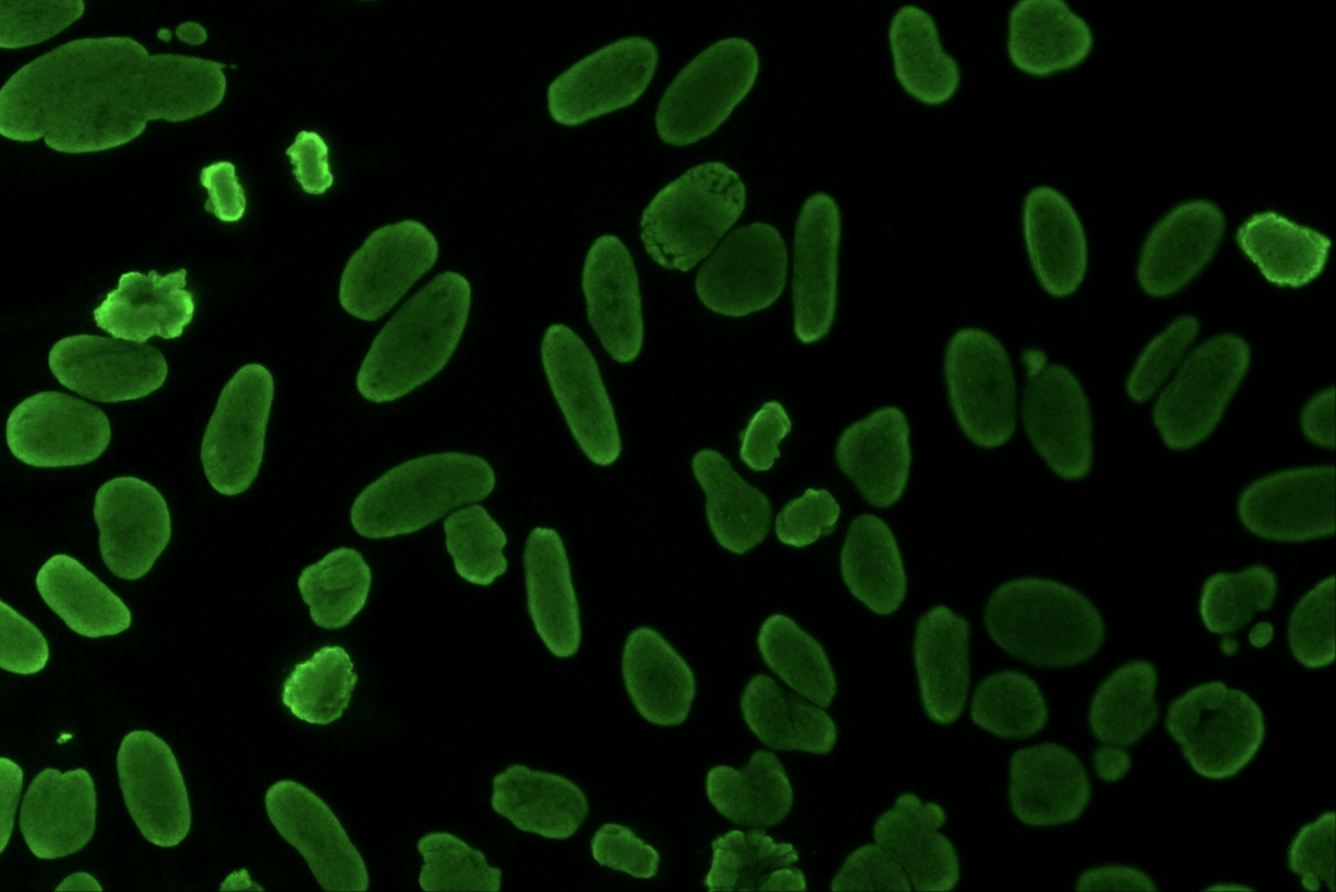
Only ANA positivity in HEp-2 cell line under immunofluorescence microscope ANA: antinuclear antibody, HEp-2: human epithelial cells

Figure 8 presents only ANA in the HEp-2 cell line. Here, fluorescence is observed coming exclusively from the nucleus of the cell. Examining the individual cells, the cytoplasm cannot be identified; only the approximately oval-shaped nucleus is prominent in the field. In particular, some cells in this field exhibit fluorescence solely from the condensed chromosomes, suggesting a homogeneous pattern of ANA.

## Discussion

Detection of specific patterns of autoantibodies against various cellular components aids in the diagnosis of the different spectrums of SARDs. These patterns include both ANA and anticytoplasmic antibodies. Our study observed several cases (6.5%) positive for isolated anticytoplasmic antibodies among patients with autoimmune disorders who were negative for ANA. This finding also highlighted a female predominance (87%) and showed that the reproductive age group was the most affected (58%).

Our study demonstrated ACA positivity in 60% of the selected diagnosed or suspected SARD patients. Among these, 6.5% had isolated anticytoplasmic antibodies, 87% had isolated ANA, and 17.4% exhibited a combination of ANA and anticytoplasmic patterns. Chan et al. described different patterns of autoantibodies targeting various cellular components and proposed a classification table based on HEp-2 cell patterns [22]. However, no universally accepted terminology has been established to differentiate these autoantibodies, contributing to diagnostic confusion in SARD patients.

Among the nuclear patterns, fine-speckled patterns were predominant (39.6%), followed by coarse-speckled (19.5%) and homogeneous patterns (6.1%). These findings differ slightly from those of Sebastian et al. [23], Akmatov et al. [24], and Sener et al. [25], but align with results reported by Mengeloglu et al. [26], Minz et al. [5], and Peene et al. [27], who found speckled patterns to be more common than homogeneous ones. This variation suggests that the distribution of autoantibody patterns in SARD patients differs across populations.

In a study on ANA-positive autoimmune disorders in Northern India, Minz et al. reported a rising trend in autoimmune disorders in that region, which supports our findings. They also noted a predominance of autoimmune disorders among females of reproductive age, with a shift to male predominance after the sixth decade of life. In 2012, Minz et al. identified SLE as the most common autoimmune disorder associated with ANA positivity in Northern India [5]. In contrast, our study found arthritis more predominant, followed by SLE and liver pathology among patients with isolated anticytoplasmic antibody positivity.

Our study also showed that the 21-45 age group was the most affected, corroborating data from a study conducted in Central India by Gupta et al., which also reported a higher incidence among females [6]. Consistently, our study found that 87% of positive cases occurred in females.

However, none of the previous studies have demonstrated significant findings regarding anticytoplasmic antibody-positive HEp-2 cell patterns and their disease associations. In contrast, our study identifies associations of vasculitis (13%), connective tissue disorders (13%), liver pathology (10.5%), SLE (10.5%), arthritis (10.5%), rheumatoid arthritis (8%), Sjögren's syndrome (8%), and systemic sclerosis (5%) with isolated anticytoplasmic antibody-positive cases.

Based on our detailed analysis, we conclude that this study possesses several strengths. It includes 973 subjects, a sample size sufficient to establish meaningful conclusions. Prior to this research, no organized data existed on the distribution and patterns of ANA in the sub-Himalayan population of West Bengal. Therefore, this study is among the first of its kind. In addition to detailing major ANA patterns, it presents definitive prevalence data on anticytoplasmic antibody-positive cases, supporting the utility of ACA in place of ANA for diagnosing SARD patients.

Like all studies, ours also has limitations that must be acknowledged. First, the sample was drawn from patients attending the outpatient department and inpatients across various departments of North Bengal Medical College and Hospital, West Bengal. These individuals were either suspected of or already diagnosed with SARDs, introducing a selection bias. Consequently, this study cannot be used to determine the true prevalence of isolated anticytoplasmic antibody-positive SARD patients in the northeastern population of West Bengal. Second, as all tests were performed manually, there is potential for subjective bias in identifying different patterns of ACA. All samples were tested at a 1:100 dilution; increasing the dilution up to 1:360 may yield different results. Additionally, although immunofluorescence microscopy was used to analyze serum samples, immunoblotting did not confirm the findings, which were beyond the scope of this study. Finally, none of the patients were followed up to observe potential changes in antibody patterns after treatment.

## Conclusions

Different autoantibodies are positive in immunofluorescence immune assays for the diagnosis of SARDs. The presence of isolated anticytoplasmic antibodies demonstrates that ANA-negative cases can still involve autoimmune diseases. Therefore, it is crucial to identify specific autoantibodies and correlate them with the corresponding autoimmune conditions. When antibodies outside the nucleus can predict autoimmune disease diagnoses, ACA is more appropriate than ANA for detecting and reporting SARDs to minimize false-negative results.
